# Visceral leishmaniasis in patients with lymphoma: Case reports and review of the literature

**DOI:** 10.1097/MD.0000000000022787

**Published:** 2020-11-06

**Authors:** Galith Kalmi, Marie-Dominique Vignon-Pennamen, Caroline Ram-Wolff, Maxime Battistella, Mathieu Lafaurie, Jean-David Bouaziz, Samia Hamane, Sophie Bernard, Stéphane Bretagne, Catherine Thiéblemont, Martine Bagot, Adèle de Masson

**Affiliations:** a Service de Dermatologie, APHP, Hôpital Saint Louis; b INSERM U976; c Laboratoire de Pathologie; d Département des maladies infectieuses et tropicales; e Laboratoire de Parasitologie, APHP, Hôpital Saint Louis; f Université de Paris; g Service d’Hémato-oncologie, APHP, Hôpital Saint Louis, Paris, France.

**Keywords:** diagnostic challenge, lymphoma, negative HIV status, visceral leishmaniasis

## Abstract

**Introduction:**

Non–HIV-related visceral leishmaniasis (VL) is becoming increasingly prevalent in nontropical countries because of the increasing number of patients with chronic diseases and the development of immune-modulating drugs.

**Patient concerns:**

Case 1 is a 60-year-old male patient of Senegalese origin presented with weight loss, lymphadenopathy, anemia, and elevated lactate dehydrogenases. Case 2 is a 46-year-old male patient of Algerian origin, with a negative HIV serology presented with cutaneous lesions.

**Diagnosis:**

Patient 1: The diagnosis of stage IV lymphocytic lymphoma (LL) was confirmed by an inguinal nodal biopsy in 2013. Patient 2: The diagnosis of T-cell lymphoma was made in 2003.

**Interventions:**

Patient 1 received 5 cycles of bendamustine and rituximab followed by a complete remission. Patient 2 was initially treated with >10 different treatments followed by 8 different chemotherapy regimens due to the disease progression.

**Outcomes:**

Patient 1: In 2017, after a follow-up of 4 years, the patient presented with fever, lymphadenopathy, splenomegaly, and pancytopenia in the setting of hemophagocytic syndrome. The initial diagnosis was a relapse of lymphoma and the patient was treated with ibrutinib. His status worsened, and *Leishmania* DNA was detected by polymerase chain reaction (PCR) on the blood and bone marrow aspirates. Ibrutinib was stopped. Amphotericin B treatment induced a complete clinical remission and clearance of *Leishmania* DNA from the blood.

Patient 2: In 2017, after a follow-up of 14 years, the patient presented with fever, lymphadenopathy, hepatosplenomegaly, pancytopenia with hemophagocytic syndrome, and an increase in the tumor skin lesions. A skin biopsy was taken from the face and the patient. A careful reexamination of the skin biopsy revealed the presence of *Leishmania* bodies. He was treated with 40 mg/kg liposomal amphotericin B leading to a regression of the clinical symptoms and negativation of the blood PCR.

**Conclusions:**

This case study shows that VL may be a diagnostic challenge in patients with lymphoma. Reactivation or primary infection should be considered in the differential diagnosis. The purpose of this study is to remind clinicians to think of VL in patients with systemic symptoms that could be misdiagnosed as a progression of the underlying lymphoma.

## 1 Introduction

Visceral leishmaniasis (VL) is a vector-borne parasitic disease caused by a group of protozoa belonging to the *Leishmania* genus. The parasites are transmitted to humans via the bite of the phlebotome and predominantly target the reticuloendothelial system. VL is endemic in tropical and subtropical areas, including the Mediterranean basin. International travelling has caused an increase in leishmaniasis cases in nonendemic countries, making the recognition of this infection important. Non–HIV-related VL is becoming increasingly prevalent in nontropical countries because of the increasing number of patients with chronic diseases and the exponential development of immune-modulating drugs for the treatment of auto-immune, inflammatory, and neoplastic diseases, especially hematological malignancies.^[1,2]^ VL is a life-threatening condition usually presenting with hepatosplenomegaly, chronic fever, weight loss, and pancytopenia. We present a case series and review of 11 cases of VL in the setting of lymphoid malignancy, including 2 original cases. In 7 of these cases, the clinical presentation was misleading and mimicked that of a progression of the underlying lymphoma and led to the use of chemotherapy or targeted treatment of the lymphoma.

## 2 Materials and methods

We present 2 original cases and a review of the literature. Patients have provided informed consent for the publication of their cases. All the procedures were performed in accordance with the principles expressed in the Declaration of Helsinki. According to the French legislation, ethical approval was not required for this observational, noninterventional study.

Using the PubMed database, we searched all the case reports of VL associated with lymphoma since 1988 (older available case report). The MeSH terms used for the search were: «leishmaniasis », «visceral leishmaniasis », « lymphoma », « Hodgkin lymphoma » and «chronic lymphocytic leukemia». The search was limited to studies performed in humans, published in English or French. We selected publications on the basis of their title and abstract. We screened the references of all case reports and reviewed additional cases. Other primary or secondary causes of immunodeficiency, mucosal and mucocutaneous leishmaniasis^[3,4]^ were excluded from the analysis. We extracted data from the relevant articles, including patient demographics and a detailed medication history. We documented the clinical features of the VL and of the lymphoma, including fever, weight loss, splenomegaly, skin involvement, biological parameters, histology, treatments received, and evolution.

## 3 Case reports

### 3.1 Case 1

A 60-year-old male patient of Senegalese origin presented with weight loss and lymphadenopathy. The biological analysis showed anemia (11 g/dL), elevated lactate dehydrogenases, and an IgGk monoclonal gammopathy. A sternal puncture found a lymphocytic infiltration of the bone marrow.

The computed tomography (CT) scan revealed lymphadenopathy and splenomegaly. The diagnosis of stage IV lymphocytic lymphoma (LL) was confirmed by an inguinal nodal biopsy. He received 5 cycles of bendamustine and rituximab followed by a complete remission. This treatment was complicated by several infectious complications due to hypogammaglobulinemia.

Four years later, the patient presented with fever, lymphadenopathy, splenomegaly, and pancytopenia in the setting of hemophagocytic syndrome. The initial diagnosis was a relapse of the hematological disease and the patient was treated with ibrutinib. His status worsened, and a second bone marrow aspiration was performed. *Leishmania* DNA was detected by PCR on the blood and bone marrow aspirates. A diagnosis of *L. infantum* visceral infection was made.

The patient reported travels in Gambia, Burkina Faso, Mali, and Mauritania. His HIV status was negative. Ibrutinib was stopped. Amphotericin B treatment induced a complete clinical remission and clearance of *Leishmania* DNA from the blood.

### 3.2 Case 2

**Figure 1:**
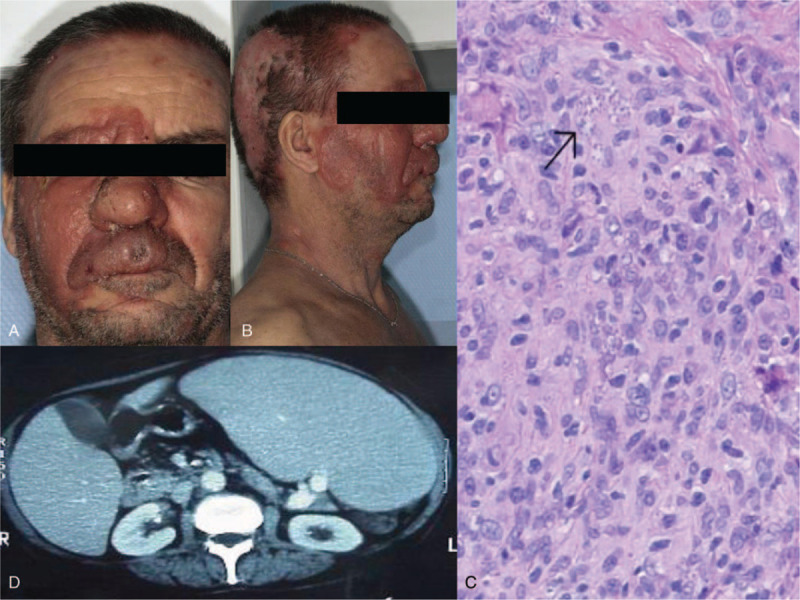
Clinical, histological, and radiological signs in a patient with cutaneous T-cell lymphoma and associated cutaneous and visceral leishmaniasis. (A, B) Clinical pictures of patient 2 with cutaneous T-cell lymphoma and associated cutaneous and visceral leishmaniasis, before treatment with amphotericin B. (C) Histology of the right cheek skin biopsy in the same patient. Hematoxylin-eosin, ×40 magnification. Histiocytes with Leishmania bodies (arrow) and lymphoma cells were coexistent on the same skin biopsy. (D) Transversal section of the computed tomography scan at the time of visceral leishmaniasis diagnosis, showing the existence of a splenomegaly.

A 46-year-old male patient of Algerian origin and with a negative HIV serology, presented with a primary cutaneous peripheral T-cell lymphoma, not otherwise specified. He was initially treated with topical steroids, PUVA therapy, interferon, bexarotene, methotrexate, followed by 8 different chemotherapy regimens due to the disease progression. Fourteen years after the initial diagnosis, he presented hemophagocytic syndrome, and an increase in the tumor lesions of the face (Fig. 1). He was then erythrodermic with splenomegaly, multiple cutaneous tumors, and fever. He was treated with systemic corticosteroids and a new line of treatment with liposomal doxorubicin. A careful reexamination of the skin biopsy performed revealed abnormally frequent histiocytes and the presence of *Leishman* bodies. *Leishmania* amastigotes were visualized on the bone marrow aspirate and *Leishmania infantum* DNA was detected by PCR in the blood and skin.^[5]^ The patient reported yearly travels to Algeria. He was treated with 40 mg/kg liposomal amphotericin B leading to a regression of the clinical symptoms and negativation of the blood PCR. Several relapses occurred requiring a maintenance treatment with amphotericin B.

## 4 Review of the literature

We identified 11 case reports of VL in the setting of lymphoma. In 2 patients, the diagnosis of lymphoma was infirmed^[6,7]^ and there was a final number of 9 patients with coexistent VL and lymphoma (Table 1).

**Table 1:** Clinical characteristics and evolution of 9 patients with lymphoma and visceral leishmaniasis from the literature.

Article	Sex	Age	Lymphoma	Clinical manifestations	Marrow infiltration	Hematologicaltreatments	Diagnosis of visceral leishmaniasis	Recent travel	Species	Leishmania serology	Leishmania on BM	Leishmaniasistreatment	Cytopenias: evolution	Blood Leishmania PCR: evolution	Evolution
Casabianca et al. Seronegative visceral leishmaniasis with relapsing and fatal course following rituximab treatment. Infection. 2011;39(4):375–8.	M	72	Follicular lymphoma	Splenomegaly and lymphadenopathy	Yes	Rituximab and polychemotherapy	After the diagnosis of lymphoma	No	Infantum	Negative	Yes	Ampho B	Persistent pancytopenia	Negativation and relapse	CR, then relapse—deceased
Cencini et al. Atypical clinical presentation of visceral leishmaniasis in a patient with non-Hodgkin lymphoma. Eur J Haematol. 2015;94(2):186.	M	60	Lymphoplasmocytic lymphoma	Splenomegaly and pancytopenia	Yes	Rituximab and bendamustine	ND	NA	NA	NA	Yes	Ampho B	Regression	NA	CR
Orlandi et al. Visceral leishmaniasis mimicking richter transformation. Leuk Lymphoma. 2014;55(12):2952–4.	M	56	Chronic lymphocyticleukemia	Splenomegaly and lymphadenopathy	Yes	Rituximab and polychemotherapy, alemtuzumab	After the diagnosis of lymphoma	NA	NA	NA	Yes	Ampho B	Regression	NA	Partial remission, then relapse
Domingues et al. Coexistence of leishmaniasis and Hodgkin's lymphoma in a lymph node. J Clin Oncol. 2009;10;27(32):e184–5.	M	15	Hodgkin lymphoma	Weightloss, fever, lymphadenopathy	None	Polychemotherapy	After the diagnosis of lymphoma	NA	Infantum	NA	Yes	Ampho B	Regression	Negativation	CR, then relapse, and second CR
Evers et al. Visceral leishmaniasis clinically mimicking lymphoma. Ann Hematol. 2014;93(5):885–7.	M	57	Splenic marginal zone lymphoma	Splenomegaly and pancytopenia	Yes	Splenectomy	Before the diagnosis of lymphoma	Spain	Infantum	Positive	Yes	Ampho B	Regression	Negativation	CR
Vase et al. Development of splenic marginal zone lymphoma in a HIV-negative patient with visceral leishmaniasis. Acta Haematol. 2012;128(1):20–2.	M	60	Splenic marginal zone lymphoma	Fever, splenomegaly and pancytopenia	Yes	Splenectomy and rituximab	Before the diagnosis of lymphoma	Mediterranean basin	Infantum	NA	NA	Ampho B	Regression	Negativation	CR
Osakwe et al. Visceral leishmaniasis with associated immune dysregulation leading to lymphoma. Mil Md. 2013;178(3):e386–9.	M	50	Angioimmunoblastic T cell lymphoma	Fever, splenomegaly, lymphadenopathy, maculopapular rash	Yes	Rituximab and polychemotherapy	After the diagnosis of lymphoma	Irak Afghanistan	NA	NA	NA	Ampho B	NA	NA	NA
Magnan et al. Visceral leishmaniasis associated with Hodgkin's disease: diagnostic difficulties. Rev Pneumol Clin. 1991;47(4):188-91.	F	19	Hodgkin lymphoma	Weight loss, fever, splenomegaly, lymphadenopathy and anemia	Yes	Polychemotherapy	Simultaneous	NA	NA	Positive	Yes	Meglumine and thenpentamidine	Regression	NA	CR
Liao et al. Concomitant T-cell prolymphocytic leukemia and visceral leishmaniasis: a case report. Medicine (Baltimore). 2018;97(38):e12410.	M	50	T-cell prolymphocytic leukemia	Weight loss, fever, splenomegaly, lymphadenopathy, skin darkening and pancytopenia	NA	NA	Simultaneous	China	Infantum	NA	Yes	Antimonium and Ampho B	NA	NA	Deceased
Case 1	M	60	Lymphocytic lymphoma	Weight loss, lymphadenopathy, splenomegaly, anemia	Yes	Rituximab and bendamustine	After the diagnosis of lymphoma	Gambia	Infantum	Positive	Yes	Ampho B	Regression	Negativation	CR
Case 2	M	46	PTCL NOS	Fever, lymphadenopathy, splenomegaly, pancytopenia	Yes	8 different chemotherapy	After the diagnosis of lymphoma	Algeria	infantym	NA	Yes	Ampho B	Regression	Negativation	CR then relapse

The median age of the patients was 56 years (range, 19–72). Eight patients were males and 1 female.

The HIV status was negative in all patients. Five patients were Europeans (Italy, Germany), 1 was Brazilian, and 1 was Japanese, the origin was unknown for the last 2 patients. Travels were reported in 4 patients (Irak and Afghanistan, n = 1; China, n = 1; Mediterranean basin, n = 1; Spain, n = 1). There were no reported data about insect bites. The hematological malignancies were Hodgkin lymphoma (HL, n = 2),^[8,9]^ splenic marginal zone lymphoma (SMZL, n = 2),^[10,11]^ follicular cell lymphoma (n = 1),^[12]^ lymphoplasmocytic lymphoma (*n* = 1),^[13]^ chronic lymphocytic leukemia (n = 1),^[14]^ angioimmunoblastic T lymphoma (n = 1),^[1]^ and T-cell prolymphocytic leukemia (T PLL, n = 1).^[15]^ All the patients had received cytotoxic chemotherapy except for the patient with T-cell prolymphocytic leukemia.^[15]^ VL developed at the time of the diagnosis of lymphoma in 2 patients,^[8,15]^ before in 2^[8,9]^ and during the course of the disease in the others.

Clinical signs and symptoms included splenomegaly in 8 patients, lymphadenopathy in 6 patients, fever in 5, weight loss in 3, and skin manifestations in 2 patients (maculopapular rash and skin darkening).

Medullary infiltration was present in 7 patients, pancytopenia in 4 patients. There was no associated hemophagocytic syndrome.

The parasitic species was *Leishmania infantum* (n = 5), and not specified in 4 cases. The diagnosis was based on medullary punction (n = 6), presence of *Leishmania* in the peripheral blood smear (n = 2) or on histology (liver biopsy, n = 1), serology (n = 3), blood PCR (n = 3), tissue PCR (kidney biopsy, n = 1). VL was mistaken as lymphoma progression in 5 patients.

The median follow-up after diagnosis was 22.8 months.(1–72)

Treatments of VL included systemic amphotericin B (n = 8), pentamidine (n = 1), antimonium (*n* = 1). The outcome was a complete remission in 4 patients with negativation of the PCR, 2 clinical complete remission of VL (with no control of the PCR), 2 patients died at last follow-up.

## 5 Discussion and conclusion

Here, we report 11 cases of VL in patients with lymphoid malignancies, including 7 cases in which VL was misdiagnosed as a progression of the underlying hematological malignancy. The expected increase in the number of international travelers and migrants, and global warming in the next decades, suggest that such cases will become more and more frequent. VL can be the result of a parasite reactivation during episodes of immune suppression, or of a recent infection after travel to endemic countries. Reactivation seems rather unlikely given the number of VL cases relative to the large number of immunocompromised patients born in endemic areas. Both our patients had recently traveled to endemic areas. Clinicians should thus be aware of the symptoms of VL and consider it in the differential diagnosis in patients with hematological malignancies. Coexistence of lymphoma and leishmaniasis in the same node was described in 2 patients with Hodgkin lymphoma.^[6,7]^ Cytokines released by recruited regulatory T cells may inhibit the immune responses to the parasite, facilitating the growth of both the tumor and parasite within the same tissue. The clinical symptoms (splenomegaly, fever, weight loss) and laboratory abnormalities (pancytopenia) are often nonspecific. Serological diagnosis of VL may be delayed or missed in patients treated with drugs that interfere with antibody production (rituximab).^[10]^ Rituximab is known to impair both antibody production and the Th2 cytokine responses by abolishing antigen presentation by B-cells, while enhancing both the number and the activity of regulatory T cells.^[10]^ Thus, blood or medullary PCR may be helpful in the diagnosis of VL and in the disease follow-up. The parasite species was *L infantum* (when specified), consistent with the fact that others species like *L major* do not disseminate.^[16]^

In 2 patients from the literature, SMZL developed after years of VL infection.^[8,9]^ There is a possible correlation between the 2 conditions because chronic antigenic stimulation by microbial agents has been proposed as a possible pathogenic mechanism in MZL. *Helicobacter pylori* infection has been involved in the pathophysiology of gastric MZL. The parasites enter the spleen and activate macrophages of the marginal zone, inducing an interleukin-10-mediated permissive environment.^[17]^ This sustained antigenic stimulation triggers polyclonal B-cell proliferation. This pathway is essential for controlling B-cell proliferation^[18]^ and its persistent activation is known to increase the risk of B cell malignancies.

In conclusion, *Leishmania* infection should be considered in the differential diagnosis of lymphoma progression in patients living in or migrating from endemic countries, presenting with fever of unknown origin, and a blood *Leishmania* PCR systematically performed in suspicious cases.

## Author contributions

**Original drafting of the manuscript:** GK, AdM

**Study supervision:** SBr, CT, MBag, AdM

**Acquisition and analysis of data:** GK, MDVP, CRW, MBat, ML, JDB, SH, SBe

**Critical review of the manuscript and final approval:** all authors

## References

[1] Osakwe NM Paulus A Haggerty PF Visceral leishmaniasis with associated immune dysregulation leading to lymphoma. Mil Med 2013;178:e386–9. 23707131 10.7205/MILMED-D-12-00407

[2] van Griensven J Carrillo E López-Vélez R Leishmaniasis in immunosuppressed individuals. Clin Microbiol Infect 2014;20:286–99. 24450618 10.1111/1469-0691.12556

[3] Yemen M Ahmed A-K Chair, Regional Leishmaniasis Control Center (RLCC). Cutaneous T cell lymphoma (CTCL) superimposed on disseminated cutaneous leishmaniasis (DCL) in an immunocompromised female from Yemen. Int J Clin Dermatol Res 2017;4–8.

[4] Nicodemo AC Duailibi DF Feriani D Mucosal leishmaniasis mimicking T-cell lymphoma in a patient receiving monoclonal antibody against TNFα. PLoS Negl Trop Dis 2017;11:e0005807. 28934199 10.1371/journal.pntd.0005807 PMC5608166

[5] Foulet F Botterel F Buffet P Detection and identification of leishmania species from clinical specimens by using a real-time PCR assay and sequencing of the cytochrome b gene. J Clin Microbiol 2007;45:2110–5. 17475750 10.1128/JCM.02555-06 PMC1932983

[6] Kawakami A Fukunaga T Usui M Visceral leishmaniasis misdiagnosed as malignant lymphoma. Intern Med 1996;35:502–6. 8835605 10.2169/internalmedicine.35.502

[7] Cohen D Fields S CT findings in visceral leishmaniasis mimicking lymphoma. Comput Med Imaging Graph 1988;12:325–7. 3179987 10.1016/0895-6111(88)90044-4

[8] Magnan A Jacquème P Sudan N Visceral leishmaniasis associated with Hodgkin's disease: diagnostic difficulties. RevPneumol Clin 1991;47:188–91. 1723216

[9] Domingues M Menezes Y Ostronoff F Coexistence of Leishmaniasis and Hodgkin's lymphoma in a lymph node. J Clin Oncol 2009;27:e184–5. 19805673 10.1200/JCO.2009.22.7835

[10] Vase MØ Hellberg YK Larsen CS Development of splenic marginal zone lymphoma in a HIV-negative patient with visceral leishmaniasis. Acta Haematol 2012;128:20–2. 22572474 10.1159/000337341

[11] Evers G Pohlen M Berdel WE Visceral leishmaniasis clinically mimicking lymphoma. Ann Hematol 2014;93:885–7. 24061787 10.1007/s00277-013-1896-9

[12] Casabianca A Marchetti M Zallio F Seronegative visceral leishmaniasis with relapsing and fatal course following rituximab treatment. Infection 2011;39:375–8. 21538038 10.1007/s15010-011-0109-5

[13] Cencini E Lazzi S Fabbri A Atypical clinical presentation of visceral leishmaniasis in a patient with non-Hodgkin lymphoma. Eur J Haematol 2015;94:186. 24635670 10.1111/ejh.12312

[14] Orlandi EM Malfitano A Visceral leishmaniasis mimicking Richter transformation. Leuk Lymphoma 2014;55:2952–4. 24576166 10.3109/10428194.2014.897704

[15] Liao H Jin Y Yu J Concomitant T-cell prolymphocytic leukemia and visceral leishmaniasis: a case report. Medicine (Baltimore) 2018;97:e12410. 30235714 10.1097/MD.0000000000012410 PMC6160114

[16] Foulet F Leishmania major cutaneous leishmaniasis in HIV-positive patients does not spread to extralesional sites. Arch Dermatol 2006;142:1361. 17043205 10.1001/archderm.142.10.1368

[17] Kopterides P Mourtzoukou EG Skopelitis E Aspects of the association between leishmaniasis and malignant disorders. Trans R Soc Trop Med Hyg 2007;101:1181–9. 17870139 10.1016/j.trstmh.2007.08.003

[18] Mendoza H Ardaiz MA Sánchez-Alvarez J Leishmaniasis with hypogammaglobulinemia and its relationship with T-cell lymphoma. EnfermInfeccMicrobiol Clin 1997;15:226–7. 9312287

